# Translating research into policy and practice in developing countries: a case study of magnesium sulphate for pre-eclampsia

**DOI:** 10.1186/1472-6963-5-68

**Published:** 2005-11-01

**Authors:** Morten Aaserud, Simon Lewin, Simon Innvaer, Elizabeth J Paulsen, Astrid T Dahlgren, Mari Trommald, Lelia Duley, Merrick Zwarenstein, Andrew D Oxman

**Affiliations:** 1 Norwegian Knowledge Centre for Health Services, Box 7004 St. Olavs Plass, N-0130 Oslo, Norway; 2 Department of Public Health and Policy, London School of Hygiene and Tropical Medicine, Keppel Street, London WC1E 7HT, UK; 3 Health Systems Research Unit, Medical Research Council of South Africa, South Africa; 4 Directorate for Health and Social Affairs, Postbox 7000 St. Olavs plass, N-0130 Oslo, Norway; 5 Department of Psychiatry and Behavioural Sciences, 15 Hyde Terrace, Leeds LS2 9JT, UK; 6 Institute for Clinical Evaluative Sciences, University of Toronto, G1 06, 2075, Bayview Avenue, Toronto, ON, Canada M4N 3M5

## Abstract

**Background:**

The evidence base for improving reproductive health continues to grow. However, concerns remain that the translation of this evidence into appropriate policies is partial and slow. Little is known about the factors affecting the use of evidence by policy makers and clinicians, particularly in developing countries. The objective of this study was to examine the factors that might affect the translation of randomised controlled trial (RCT) findings into policies and practice in developing countries.

**Methods:**

The recent publication of an important RCT on the use of magnesium sulphate to treat pre-eclampsia provided an opportunity to explore how research findings might be translated into policy. A range of research methods, including a survey, group interview and observations with RCT collaborators and a survey of WHO drug information officers, regulatory officials and obstetricians in 12 countries, were undertaken to identify barriers and facilitators to knowledge translation.

**Results:**

It proved difficult to obtain reliable data regarding the availability and use of commonly used drugs in many countries. The perceived barriers to implementing RCT findings regarding the use of magnesium sulphate for pre-eclampsia include drug licensing and availability; inadequate and poorly implemented clinical guidelines; and lack of political support for policy change. However, there were significant regional and national differences in the importance of specific barriers.

**Conclusion:**

The policy changes needed to ensure widespread availability and use of magnesium sulphate are variable and complex. Difficulties in obtaining information on availability and use are combined with the wide range of barriers across settings, including a lack of support from policy makers. This makes it difficult to envisage any single intervention strategy that might be used to promote the uptake of research findings on magnesium sulphate into policy across the study settings. The publication of important trials may therefore not have the impacts on health care that researchers hope for.

## Background

Translating research evidence into policy is crucial to improving the evidence base of health care as well as to improving health care outcomes. Indeed, the recent Ministerial Summit on Health Research in Mexico City noted that, "Research has a crucial but under-recognised part to play in strengthening health systems, improving the equitable distribution of high quality health services, and advancing human development." [1]. Other have voiced similar views [2-4]. However, knowledge regarding both the factors affecting the use of evidence by policy makers and the effectiveness of interventions to improve the use of evidence by policy makers remains underdeveloped [5,6]. While some studies have been undertaken in developing countries [7,8], knowledge from these settings is particularly thin. For example, a recent systematic review of interview studies and surveys of health policy makers' perceptions of their use of evidence found 24 eligible studies, of which only four were conducted in low and middle income country settings [5]. Further studies of research-policy linkages in developing countries could therefore be helpful.

A number of models have been suggested to explain the role of research in policy making [9-13]. In summary, the models include so-called 'rational' approaches in which the uptake of research findings into policy making is envisaged as a linear process, with research-based knowledge promoting policy change and practice. Alternative approaches view the research-policy relationship as one of 'enlightenment' suggesting, in contrast to the linear models, that research findings 'percolate' through the policy environment, gradually influencing ideas and approaches. These approaches suggest a more complex and contested relationship between research and policy making, with research being one of many knowledge sources used by policy makers in taking decisions. Others have focused on the networks of influence and 'policy communities' – including civil servants, civil society organisations and others – that may form around, and influence, specific issues. It has also been suggested that research may be used strategically to delay or support policy decisions. We return to these models later in discussing the study results.

In this paper, we report the findings of a study that examined, firstly, the factors perceived as limiting the use of the results of a recently published trial of magnesium sulphate for the treatment of pre-eclampsia and, secondly, the extent to which it might be possible to make generalisable inferences regarding barriers to and facilitators of evidence-based pharmaceutical policies for maternal and child health.

Eclampsia is an important contributor to maternal morbidity and mortality in low-income countries [14]. Eclampsia is the occurrence of a convulsion (fit) in association with pre-eclampsia. Pre-eclampsia has been defined as "a multisystem disorder [of pregnancy] that is usually associated with raised blood pressure and proteinuria but, when severe, can involve the woman's liver, kidneys, clotting system, or brain. The placenta is also often involved, with an increased risk of poor growth and early delivery for the baby." [15] An estimated 50,000 women die annually following eclamptic convulsions and 99% of these deaths occur in low and middle income countries [16]. Strong evidence of the effectiveness of magnesium sulphate for women with eclampsia has been available since 1995 [15,17,18]. Until recently, however, there was little reliable evidence regarding the effectiveness of magnesium sulphate for preventing the onset of eclampsia for women with pre-eclampsia. The results of the Magpie (MAGnesium sulphate for Prevention of Eclampsia) Trial, published in 2002, provide convincing evidence that magnesium sulphate is also effective for prevention of eclampsia [19,20]. Given this evidence, there is concern that this effective, safe and inexpensive drug may still not be available in many countries for women with eclampsia or pre-eclampsia [21,22]. Hundreds of thousands of women could benefit from these research results, provided they are translated into appropriate policies and practice. Given these concerns, this study examined factors that might hinder or facilitate the translation of the results of the Magpie Trial into appropriate policies and practice. We also discuss what might be done at international level by researchers, the WHO and other international agencies to address these.

## Methods

A case study approach, that involves examining a naturally occurring case or cases, was used to explore the research questions [23]. The selected case – in this instance how the findings of the Magpie Trial might be translated into policy in developing countries – was viewed as a whole, allowing processes to be compared and explored [24,25]. As such, we did not aim to investigate the fine detail of specific elements of the research translation process in each setting, but rather attempted to assemble a broad overview of the research-policy interface for the research finding of interest. For the purposes of this study, we understood 'policy' to include both service policies, which relate to resource allocation and the organisation of services, and practice policies, which are concerned with the use of resources by clinicians involved in delivering health care ([26]).

Data were collected in two phases using a range of complementary qualitative and quantitative methods. Firstly, we conducted observations, a semi-structured group interview and a survey of members of the Magpie Trial Collaborative Group – a research team representing 33 countries and involved in the Magpie Trial. Secondly, we conducted a survey of drug information officers in twelve developing countries. The details of these methods are described below.

### Phase 1: perceptions of the Magpie Trial collaborative group

A meeting of the Magpie Trial collaborators in March 2002 to discuss the trial findings prior to publication provided an opportunity to explore the perceptions of this group, with a strong interest in developing the evidence based for obstetric care, regarding the barriers and facilitators to implementing the findings of their study. Limited time was available during this meeting for data collection. However, we saw this opportunity as important since most of the trial collaborating centres were represented. Several data collection methods were used, as described below.

#### Observations

Small group and plenary discussions on the dissemination and implementation of the trial results were observed during the Magpie Trial meeting. The collaborators planned these independently of our study. The groups were organised by geographical regions: Africa, Asia, Latin America, and a fourth group that included European, North American and Australian collaborators. Notes of the discussions were made.

#### Group interview

A brief semi-structured group discussion was conducted with six collaborators from Brazil, Egypt, Pakistan and Uganda, purposively selected to represent a variety of developing countries. This focused on the implications of the possible Magpie Trial results and strategies for dissemination and implementation. Detailed notes of the interview were taken and the data from the interview and the observations were coded and subjected to thematic analysis. The issues that emerged were used to help interpret the survey results.

#### Survey of the Magpie Trial collaborative group

A questionnaire was designed to explore barriers and facilitators to the uptake of the Magpie Trial findings into national, regional and hospital level policies in the respondent's country. A mix of open- and closed-ended questions addressed current policies regarding magnesium sulphate, specific barriers and facilitators and necessary changes in policies. It also attempted to identify key policy makers. The questionnaire was distributed after presentation of the Magpie Trial results. Spanish speaking participants were given the option of answering the open-ended questions in Spanish. These responses were later translated into English. MA and SI independently coded and qualitatively analysed the responses to the open ended questions. Other data were entered into an Excel database for analysis.

### Phase 2: Survey of national drug information officers

Following analysis of Phase 1 data, a pilot survey of national drug information officers in twelve low and lower-middle income countries (Albania, Armenia, Bolivia, Cambodia, India, Indonesia, Iran, Nicaragua, the Philippines, Rwanda, South Africa, and Yemen) was conducted. The survey had the following objectives: to explore the generalisability of the perceptions of the Magpie Trial Collaborative Group; to collect data for countries that had not participated in the Magpie Trial (Armenia, Bolivia, Cambodia, Indonesia, Iran, Nicaragua, Philippines, Rwanda); to overcome some of the limitations of the information collected from the collaborators; to explore the feasibility of a survey approach for collecting data from a larger sample of countries; and to assess the reliability of the information gathered. The countries were purposively selected to represent geographic, cultural and political variety.

Drug information officers were identified through the World Health Organisation (WHO) Department of Essential Drugs and Medicines. We attempted to validate the information provided by drug information officers through surveying obstetricians in each country. These were identified by personal contacts. The questionnaire for drug information officers focused on the registration, supply and distribution of magnesium sulphate. Information was also sought on five other drugs: folic acid to prevent anaemia and the risk of fetal malformation, ergometrin to decrease post partum haemorrhage, oxytocin to induce labour, hydralazine to treat hypertension during pregnancy, and nevirapine to prevent mother-to-child transmission of HIV [27]. The first three of these are similar to magnesium sulphate in that they are inexpensive, have been used for many years, and indications for their use are common. Hydralazine was selected because it was being considered for removal from the model essential medicines list. Nevarapine was selected because it was expensive and relatively new. The questionnaire for obstetricians focused on the availability and use of magnesium sulphate. Both questionnaires consisted of closed-ended questions with space for comments. Data were entered into an Excel database and analysed using simple descriptive statistics.

## Results

### Group interview and observations – Magpie Trial collaborators

The group interview and observations identified a number of issues seen by Magpie collaborators as barriers to translating the Magpie Trial results into appropriate policies and practice. In presenting these, we have utilised the policy analysis framework of actors, context, content and process as an organising scheme [28]. With regard to context, a range of problems with the availability of magnesium sulphate across the trial settings was discussed. The drug was reported to be available in Latin America, Bangladesh and to some extent in India. In Pakistan and Uganda, however, magnesium sulphate was not registered as a pharmaceutical and respondents noted that there was controversy over whether it should be included in the essential medicines list. Access was also seen as a problem in Zimbabwe as the drug had not been registered even though it was listed on the essential medicines list. One participant commented that magnesium sulphate "is so cheap that the pharmaceutical companies don't bother to push for its registration", indicating one reason for the failure to register the drug in some settings. In contrast, access to magnesium sulphate was not considered to be a problem in high income settings and very frequent use was identified as a problem in parts of Latin America. There was a range of views regarding the potential for local manufacture of magnesium sulphate – a route seen as potentially useful in ensuring a low cost supply of the drug. Other problems raised included inequalities in availability between government and private hospitals and patient level barriers to improving attendance for antenatal care, including cost, transportation, traditions and beliefs. It was suggested that policy makers and politicians needed to be brought "on board" with health care professionals to address these issues.

A number of actors were identified as important to the research translation process. The collaborators suggested that few clinicians or policy makers in their settings were aware of the concept of evidence-based medicine, would read the Magpie Trial report or be able to interpret the findings. Even if clinicians were aware of the Magpie Trial results, few had opportunities to influence relevant policies. Collaborators from West Africa felt that there was a need, through medical associations, to involve politicians at the national level in discussing the trial findings. Such policy makers were seen to be distant from poor and under-resourced areas of the country, further exacerbating barriers to the development and application of appropriate policies for these areas. Collaborators from high income countries also thought that professional organisations would be important in drawing out the implications of the trial findings and making clear recommendations.

The collaborators held differing views about the importance of support from WHO and the United Nations Children's Fund (UNICEF) in influencing policies and practice. For example, collaborators from Nigeria considered it important to get support from the WHO region as this office had close relations with policy makers and politicians. One collaborator in the Asian group argued that if WHO provided magnesium sulphate to health facilities at no cost for one year, this would create pressure on governments and pharmaceutical companies to continue supplying it thereafter. In at least one of the countries represented in the discussion, UNICEF was attempting to implement protocols for emergency obstetrical care and was therefore seen as having as import role in policy making. The influence of international agencies, such as WHO and UNICEF, on the mass media was also highlighted.

A range of problems with the process of developing and implementating clinical guidelines for the treatment of hypertensive disorders of pregnancy was raised. Some countries had no national guidelines and institutions were not under any obligation to follow recommended procedures. Consequently, each hospital followed its own policies. Elsewhere national guidelines existed, but magnesium sulphate was used routinely only for eclampsia and not for pre-eclampsia. Furthermore, even where magnesium sulphate was widely available, appropriate hospital facilities, such as intensive care units, were seen as necessary to implementing the Magpie Trial results due to the perceived need for close monitoring of patients. Several participants suggested that an active guideline implementation process was required at each hospital. For example, in South Africa, where national policy recommended the use magnesium sulphate for eclampsia, the main problem was considered to be training for health professionals. Training was also seen as important in the Asian countries. However, it was acknowledged that this would require substantial resources.

The content and focus of the policy implementation process was also seen as important and to require tailoring to specific settings. In Latin America one of the important issues identified was the very frequent use, rather than the underuse, of magnesium sulphate. Here, it was suggested, training and implementation needed to focus on raising awareness of the lack of evidence to support giving magnesium sulphate to women with relatively mild disease. A lack of accountability of obstetricians was also considered to be a key problem in several settings. It was suggested that legislation was needed to specify standards of, and responsibilities for care.

A number of dissemination and implementation strategies for translating the Magpie Trial findings were suggested. The African group considered it important for the researchers themselves to publish articles in a variety of national and regional medical journals to gain support for developing and implementing appropriate guidelines. Some participants felt that the mass media could play a role in advocating for the registration of magnesium sulphate, but there were perceived problems with the quality of health care reporting and a need for health care professionals to work closely with journalists. It was also noted that many journalists were based in well resourced central urban areas and did not reflect the views of more poorly resourced peripheral regions.

In summary, significant regional and national differences in the policy making context were identified, including the importance of clinical guidelines and availability of and access to magnesium sulphate. The role and influence of different policy actors, including international agencies and the media, was also seen to vary between settings, as did the barriers to the process of developing and implementing evidence based policies.

### Survey of the Magpie Trial collaborative group

A response rate of 81% (89/110 participants) was achieved. Some questions were not answered by some respondents and other responses were illegible. Table 1 shows the countries represented by the 89 respondents. The distribution reflects that of the Magpie Trial participants, with half of the respondents coming from just three countries – South Africa, Argentina and the United Kingdom – from where half of trial participants were recruited [19]. Sixty-five (73%) of the respondents were obstetricians. There were twelve "other" physicians, five midwives, two researchers, two health managers and three respondents who did not state their profession. Based on World Bank country classifications [29], 39 (44%) of the respondents represented 13 low or lower-middle income countries, 20 (22%) represented 3 upper-middle income countries, 24 (27%) represented 8 high-income countries and 6 (7%) did not state which country they represented.

**Table 1:** Geographical distribution of the respondents to the Magpie Trial Collaborative Group survey

**Region**	**Country**	**Number of respondents**
Africa		27
	South Africa	13
	Nigeria	6
	Uganda	3
	Egypt	1
	Ghana	1
	Malawi	1
	Sierra Leone	1
	Zimbabwe	1
Latin America		20
	Argentina	16
	Brazil	2
	Mexico	2
Asia		13
	India	5
	Pakistan	3
	Bangladesh	2
	Singapore	1
	UAE	1
	Yemen	1
Europe		19
	UK	16
	Albania	1
	Italy	1
	Netherlands	1
North America		2
	Canada	1
	USA	1
Australia	Australia	2
Not stated		6
Total	24	89

Table 2 shows the main barriers identified by respondents to the dissemination and implementation of the Magpie Trial results. Respondents from low and lower-middle income countries most frequently identified "political barriers" as a factor that might hinder the dissemination and implementation of the trial results (8/13 countries including 13/39 respondents). Issues cited by these respondents included lack of political will; that policy makers were poorly informed of, and insufficiently involved in, these matters; and that they did not see pre-eclampsia as a priority health problem. Public authorities were also identified as a barrier (4/13 countries including 6/39 respondents). In addition, lack of availability of the drug and of appropriate health personnel for its administration, as well as costs, were frequently raised. Respondents from two of the three upper-middle income countries (Argentina, Brazil and Mexico) highlighted political barriers (5/20 respondents) such as a lack of political engagement, lack of information or awareness (5/20 respondents), and costs (4/20 respondents) as important obstacles. A number of respondents from high-income countries indicated that applying the results of the Magpie Trial was not an important issue in their setting.

**Table 2:** Barriers to the dissemination and implementation of the results of the Magpie Trial results by country group*

**Barrier**	**Number of countries from which at least one representative gave a response**			
	
	**Low and lower-middle income countries (n = 13^#^)**	**Upper-middle income countries (n = 3^#^)**	**High-income countries (n = 8^#^)**	**Total**
Political barriers	8	2	2	12
Lack of information or awareness regarding magnesium sulphate	6	2	1	9
Costs of treatment	5	2	1	8
Lack of availability of personnel and hospitals	5	1	1	7
Lack of support from public authorities	4	1	2	7
Lack of availability of magnesium sulphate	5	0	1	6
Lack of clinical practice guidelines	1	1	3	5
Magnesium sulphate not registered for treatment of eclampsia/pre-eclampsia	1	1	0	2

* 17 respondents either did not reply or had illegible responses to this question.

^# ^Total number of countries in this group.

Table 3 shows factors identified by respondents as having the potential to facilitate dissemination and implementation of the Magpie Trial results. In low and lower-middle income countries as well as upper middle-income countries, the most frequently mentioned facilitator was establishing channels to public authorities (6/13 and 2/3 countries respectively). In addition, clinical practice guidelines, resources and international organisations were seen as playing an important role in low and lower-middle income countries. Professional organisations were seen as important in upper middle-income countries (3/3 countries). The most frequently mentioned facilitator for the high-income countries was clinical practice guidelines (4/8 countries). In general, the facilitators identified addressed the barriers highlighted by the respondents.

**Table 3:** Facilitators to implementing the results of the Magpie Trial by country*

**Facilitator**	**Number of countries from which at least one representative gave a response**			
	
	**Low and lower-middle income countries ** **(n = 13^#^)**	**Upper-middle income countries ** **(n = 3^#^)**	**High-income countries ** **(n = 8^#^)**	**Total**
Channels to public authorities	6	2	2	10
Development/use of clinical practice guidelines	4	1	4	9
Publications in medical journals	5	0	2	7
Resources	4	1	1	6
International organisations	4	1	0	5
Professional organisations	2	3	3	8
Licensing and availability of magnesium sulphate	3	0	3	6

* 29 respondents provided illegible responses to this question.

^# ^Total number of countries in this group.

Representatives from 7/13 low and lower-middle income countries and 2/8 high-income countries reported that magnesium sulphate was not licensed for the treatment of pre-eclampsia (not shown in tables). This barrier was not raised by respondents in upper-middle income countries. However, there were frequently conflicting responses to this question by respondents from same country. Reasons why magnesium sulphate was not licensed included failure to submit an application for licensing and "red tape". In each of 4/13 low and lower-middle income countries, one or more respondents said that magnesium sulphate was not available. Distribution of magnesium sulphate was reported to be a problem by at least one respondent from 8/13 low and lower-middle income countries and 1/3 upper-middle income countries.

Respondents indicated that there were no national clinical practice guidelines for pre-eclampsia in 8/13 low and lower-middle income countries and 2/8 high-income countries. Local (hospital) guidelines were available in most settings (82% of respondents). However, only half of respondents considered the recommendations in local guidelines to be appropriate in light of the Magpie Trial results.

Contextual health system factors were identified by respondents as important in determining the uptake of the trial findings into policy. At least one respondent in 13/16 low and middle income countries noted that fewer than 60% of pregnant women had access to services where pre-eclampsia was likely to be appropriately diagnosed. Furthermore, fewer than 40% of pregnant woman had access to professionals who could administer magnesium sulphate, according to at least one respondent in 10/13 low and lower-middle income countries. The costs of magnesium sulphate and hospital care were thought likely to hinder the dissemination and implementation of the Magpie Trial results in six of these countries. However, there were frequently different responses to this question from representatives of the same country. At least one respondent from ten of the thirteen low and lower-middle income countries thought that costs were not a barrier.

Most of the respondents from low and lower-middle income countries considered it important to change policies in their countries to improve the availability (median importance score of 5.0 on a scale from 1 [not important] to 5 [very important]) and distribution of magnesium sulphate (median importance score = 5.0). Improving access to hospitals offering treatment with magnesium sulphate and to professionals trained to manage pre-eclampsia were also seen as important (median importance scores = 5.0 and 5.0 respectively). Respondents from upper-middle income countries saw changes in policies to improve the availability of the drug as less important (median score = 2.5). The other three issues (drug distribution, access to hospitals and access to trained professionals) were seen as important by these respondents (median importance scores = 4.0, 4.0 and 5.0). For high income countries, however, all four of these issues were perceived to be less crucial (median importance scores = 1.0, 1.0, 1.0 and 2.0). Changes in clinical practice guidelines were seen as necessary in low and high-income countries (median importance 5.0 and 4.8 respectively), but less so in upper-middle income countries (median importance 3.5). Changes in policies regarding payment for magnesium sulphate and hospital costs were considered more important in low and lower-middle income countries (median importance 5.0 and 5.0 respectively) than in upper-middle income countries (median importance 2.0 and 4.0) and high-income countries (median importance 1.0 and 1.0). Responsibility for payment for magnesium sulphate and hospital care for pre-eclampsia varied across and within countries and included governments, patients and private insurance.

A range of professional and organisational actors was seen to be influential in changing relevant policies related to the use of magnesium sulphate (Table 4). A majority of respondents from low and middle-incomes countries considered central health authorities and hospital administrations to be influential. This contrasted with high-income countries where these groups were less frequently seen as having an important role in policy change. There were important differences between low and lower-middle income countries and upper middle-income and high income countries regarding the perceived influence of the WHO, drug licensing agencies, the pharmaceutical industry, politicians and patient organisations. Notably, only a minority of respondents considered patient organisations to be influential (18% overall).

**Table 4:** Organisations and individuals noted by respondents as having an important influence on changing policies related to the use of magnesium sulphate in their countries (%)

**Organisation or individuals**	**Low and lower-middle income countries** **(n = 39^#^)**	**Upper-middle income countries** **(n = 20^#^)**	**High-income countries** **(n = 24^#^)**
Medical or obstetrical association	92%	85%	88%
Hospital department of obstetrics	87%	85%	83%
Central health authorities	82%	70%	38%
Hospital administration	79%	60%	17%
World Health Organisation	79%	45%	8%
Nurse or midwife association	72%	35%	58%
Drug licensing agency	72%	10%	46%
Pharmaceutical industry	69%	25%	33%
Regional or local health authorities	67%	90%	33%
Mass media	56%	20%	46%
Individual influential professionals	51%	55%	75%
Politicians	51%	55%	8%
Public health insurance program	26%	65%	13%
Private health insurers	26%	35%	8%
Non-governmental organisations	26%	15%	4%
Other international organisations	26%	15%	4%
Other professional associations	21%	15%	17%
Patient organisations	13%	10%	33%
Others	13%	25%	21%

# Number of respondents.

### Survey of drug information officers

Following repeated efforts, responses were obtained from national drug information officers or drug regulatory officials for only nine of the twelve countries. Their responses regarding the licensing, supply and distribution of magnesium sulphate are shown in Table 5. In contrast to the survey of Magpie collaborators, these respondents did not report problems with licensing, supply or distribution in their countries, and noted that magnesium sulphate was on the essential medicines list for seven of the nine sampled.

**Table 5:** Licensing, supply and distribution of magnesium sulphate as reported by drug information officers or drug regulatory officials – survey

**Country**	**Licensed for eclampsia**	**Licensed for pre-eclampsia**	**Imported or produced locally**	**Problems with supply or distribution**	**MgSO4 on EML**	**Date included on EML**
Armenia	Yes	Yes	Imported	No	Yes	1994
Bolivia	Yes	Yes	Both	No	Yes	1985
Cambodia	Yes	Yes	Imported	No	Yes	2000
India	*NR*	*NR*	Produced locally	No	No	*NA*
Indonesia	Yes	Yes	Produced locally	No	Yes	1983
Iran	Yes	Yes	Produced locally	No	Yes	1981
Philippines	Yes	*NR*	Produced locally	No	Yes	1989
Rwanda	Yes	Yes	Both	No	Yes	*NR*
Yemen	Yes	Yes	Imported	No	No	*NA*

MgSO4 = magnesium sulphate

EML = essential medicines list

NR = not reported

NA = not applicable

Obstetricians in five of these nine countries (Armenia, India, Indonesia, the Philippines and South Africa) suggested that magnesium sulphate was widely available and widely used for both eclampsia and pre-eclampsia (Table 6). However, there was some uncertainty regarding the importance of limitations to availability and differences in availability across geographical areas and between public and private hospitals.

**Table 6:** Availability of magnesium sulphate and use for eclampsia and pre-eclampsia as reported by obstetricians – survey

	**Eclampsia**					**Pre-eclampsia**	
	
**Country**	**Available** **in hospitals**	**Geographic differences** ** in availability**	**Public vs. Private** ** hospitals**	**Used for** **women with eclampsia**	**Reasons for MgSO4 not** ** being used for all women**	**Used for women with** ** pre-eclampsia?**	**Reasons for MgSO4 not** ** being used for all women**
**Armenia**							
Obstetrician 1	All	No	No	All		All	
Obstetrician 2	Most	No	No	All		All	
Obstetrician 3	Most	No	No	All		All	
**India**							
Obstetrician 1	All	No	No	All		All	
Obstetrician 2	Some	Yes	No	Most		Few	D, E
Obstetrician 3	Some	Yes	No	Some	A	Some	E
Obstetrician 4	Some	Yes	No	Most		Some	D, E
**Indonesia**							
Obstetrician 1	Most	Yes	No	Most	A, B, C	Most	D, E
Obstetrician 2	Some	Yes	Yes	Most	A	Most	D, E
**Philippines**							
Obstetrician 1	Most	Yes	Yes	All		All	
Obstetrician 2	All	Don't know	Don't know	All		All	
**South Africa**							
Obstetrician 1	All	No	No	All		Most	
Obstetrician 2	All	No	No	All		Some	F

A = Problems with availability

B = Different drug used

C = Lack of awareness among clinicians

D = Lack of awareness among clinicians

E = Problems with availability

F = Not a priority

MgSO4 = magnesium sulphate

No problems were reported by drug information officers regarding the registration, supply or distribution of folic acid in any of the nine countries, with the exception of Bolivia. The same was true for ergometrin, with the exception of Cambodia, where frequent shortages were reported due to its short shelf life. No problems were reported for oxytocin (Table 7). This contrasts with magnesium sulphate for which numerous problems with supply and distribution were noted.

**Table 7:** Licensing, supply and distribution of five other obstetrical drugs as reported by drug information officers or drug regulatory officials – survey

**Country/drug**	**Licensed**	**Problems with supply or distribution**	**Comments**
**Armenia**			
Folic acid	Yes	No	
Ergometrin	Yes	No	The registered form is methylergometrine.
Oxytocin	Yes	No	
Hydralazine	No	Yes	There is a demand for the drug, but no interest from drug companies.
Nevirapine	Yes	Yes	There is a demand for the drug, but no interest from drug companies.
**Bolivia**			
Folic acid	Yes & No	Yes	Availability problems within the public health services (logistical).
Ergometrin	No	No	
Oxytocin	No	No	
Hydralazine	No	Yes	Not available in the national market. Only imported by two suppliers for use in public health facilities.
Nevirapine	Yes	Yes	Not on national EML & not registered so not available on the national market. AZT is available but expensive.
**Cambodia**			
Folic acid	Yes	No	
Ergometrin	Yes	Yes	There are often shortages due to short shelf-life.
Oxytocin	Yes	No	
Hydralazine	Yes	Yes	There are often shortages due to short shelf-life.
Nevirapine	No	*DN*	Not available in Cambodia.
**India**			
Folic acid	*NR*	No	
Ergometrin	*NR*	No	
Oxytocin	*NR*	No	
Hydralazine	*NR*	Yes	Low demand as a better therapeutic alternative is available. Produced by one manufacturer only.
Nevirapine	*NR*	Yes & No	
**Indonesia**			
Folic acid	No	No	
Ergometrin	No	No	
Oxytocin	No	No	
Hydralazine	No	No	
Nevarapine	*DN*	*DN*	Only been registered in 2002 so too early to know if there are any problems with supply.
**Iran**			
Folic acid	Yes	No	
Ergometrin	Yes	No	
Oxytocin	Yes	No	
Hydralazine	Yes	No	
Nevirapine	No	Yes	This drug is not being used in Iran.
**Philippines**			
Folic acid	Yes	No	
Ergometrin	Yes	No	
Oxytocin	Yes	No	
Hydralazine	Yes	No	
Nevirapine	No	*NR*	Not registered by the Bureau of Food & Drugs.
**Rwanda**			
Folic acid	Yes	No	
Ergometrin	*DN*	No	
Oxytocin	Yes	No	
Hydralazine	Yes	Yes	Many health facilities of out of stock.
Nevirapine	Yes	No	
**Yemen**			
Folic acid	Yes	*DN*	
Ergometrin	Yes	*DN*	
Oxytocin	Yes	*DN*	
Hydralazine	*DN*	*DN*	
Nevirapine	*DN*	*DN*	No cases.

DN = do not know

NR = not reported

For hydralazine, drug information officers in three countries reported that it was not licensed and in five countries that there were problems with its supply. Problems with the registration, supply and distribution of nevirapine were reported in four of the nine countries, with Rwanda the only country not reporting a problem with the supply or distribution of this drug. Nevirapine is an expensive drug compared to magnesium sulphate and indications for its use vary across countries.

Given the difficulties in accessing appropriate respondents for this study, and respondents' limited knowledge of the information required by the questionnaire, we decided that a larger survey of drug information officers in low and lower-middle income countries would not likely provide reliable and useful information.

## Discussion

This multi-country study used a range of methods to explore the wide variety of factors that hinder and facilitate the translation of research findings, in this case from the Magpie Trial, into policies and practice across a range of settings. It highlights two important issues: firstly, the barriers to translating the findings of studies such as the Magpie Trial into policy are complex, multifactoral and context specific, as has been suggested elsewhere [2,11]. For example, if one considers barriers within the health system, such as drug availability and licensing, this study demonstrates that the availability of magnesium sulphate remains an issue in some settings, although it was difficult to ascertain the extent of this problem and there is likely to be local variation. Furthermore, it proved difficult to ascertain the number of countries in which magnesium sulphate is not licensed for the treatment of eclampsia. Even if the availability of magnesium sulphate was assured, clinical practice guidelines and effective strategies to implement these would still be needed to address barriers at the practitioner level, and would need to be tailored to particular settings. Although there is evidence from some settings that practitioners may rapidly and extensively change practice in response to new evidence from RCTs and systematic reviews [30-33], this has not been commonly demonstrated in resource poor settings. This may reflect differences in facilitators of change between low and high income settings, as discussed earlier.

Another complex issue is that of interaction with and support from policy makers – the barrier most frequently cited by respondents. Although we had not anticipated this barrier, and had not asked about it in the closed-ended questions, this finding mirrors that of a recent systematic review of the use of research in health policy-making in which 'absence of personal contact' between researchers and policy makers was identified as the most common problem [5]. Although magnesium sulphate is relatively inexpensive in terms of purchase price – less than US$5 per patient [21] – political support and national policies may be needed for a number of reasons. These include registering the drug where it is not licensed, ensuring that it is distributed and available in hospitals, ensuring that health care professionals are appropriately trained, and facilitating the access of women with pre-eclampsia to hospital care. Given the complex nature of the barriers to translating the Magpie Trial findings into policy, the 'rational' model of the research-policy relationship, in which new knowledge results in policy change, therefore seems a less appropriate description of this research translation process than the 'enlightenment' model, which describes a more diffuse, non-linear relationship between research and policy and recognises that a single research study is unlikely to have a direct impact on policy [10].

The second key finding is the difficulties experienced in obtaining detailed information on drug availability and use at country level. The pilot survey focused on low and lower-middle income countries, where there appeared to be both the greatest need and the largest problems. Information was sought from the WHO's network of designated national drug information officers because we believed they would be able to reliably answer questions about the licensing, supply and distribution of magnesium sulphate. However, it was frequently difficult to contact these officers. When they could be reached, they had reliable but limited information about licensing, importation and local production, but no information on actual use. Beyond this there were little, if any, reliable data from WHO or other sources regarding the availability of magnesium sulphate or other drugs at country level. This suggests that a large investment of resources would be required both to ascertain the magnitude and precise nature of problems regarding the distribution and use of magnesium sulphate internationally and to explore how best to address these. The difficulties in obtaining information, combined with the wide and differing range of barriers between settings, makes it difficult to design any single, widely generalisable intervention package that might be used to promote the uptake of the Magpie Trial findings into policy and practice across the study settings. This is also likely to be the case for other drug and maternal and child health interventions that research has shown to be effective and that researchers and others wish to translate into policy and practice.

This study has several limitations. Firstly, the findings from the Magpie Trial Collaborative Group reflect the nature of that group, comprised primarily of self-selected obstetricians with a professional interest in pre-eclampsia. However, there were several reasons why this study was initiated with this Group, despite these limitations. It was their concerns about failures to translate results from their earlier trial [17] into practice in developing countries [21,22] that prompted this study. They were therefore motivated to participate in it. Furthermore, the Group had expert knowledge of both the specific topic and obstetrical care in their settings. Also, our survey of drug information officers, conducted to complement the data provided by the Group, demonstrates that it is difficult to access reliable information, reflecting in part the paucity of rigorous data regarding the availability and use of magnesium sulphate in low and middle-income countries. The data from the Collaborative Group therefore provide useful insights into the potential and limitations of senior researchers across a wide range of countries to influence the translation of their findings into appropriate health care policies.

This study attempted a broad overview of research translation across a range of countries. This approach had the advantage of allowing us to identify commonalities and differences between countries, thereby improving the generalisability of the findings, but also limited the depth of enquiry in any one setting. Although we have taken the approach of grouping country findings using the World Bank classification of level of income, we acknowledge that this approach might mask differences both within these groups and at sub-national levels. Further in-depth studies, currently in progress, are exploring the use of research evidence in policy making in several of the countries included in the Magpie Trial (G Woelk, personal communication). These studies are exploring the viewpoints of a wider group of policy actors, including senior policy makers within ministries and departments of health and representatives of international organisations.

## Conclusion

Despite robust evidence from a landmark trial and systematic review of the effectiveness of magnesium sulphate for the treatment of pre-eclampsia, the drug is still not available in some countries and its availability varies in many others. Licensing, importation and production are probably not the most important barriers in most settings to translating this research evidence into practice. Rather, a complex and multifaceted group of issues, differing across contexts, inhibits the uptake into policy of research findings on health issues such as the treatment of eclampsia and pre-eclampsia [2]. The changes that are needed to ensure widespread and equitable availability and use of magnesium sulphate are therefore also variable and complex. This helps to explain why publication of the findings of important studies, such as the Magpie Trial, are important but generally not sufficient to change policy and practice in the health services, particularly in low and middle income countries. In the meantime, many women die each year from complications of pregnancy associated with pre-eclampsia [16]. What then can be done by researchers and other actors and where does responsibility lie?

Firstly, there is a need to identify credible national advocates or "knowledge brokers"[4,6]. The Magpie Trial collaborators are willing advocates but frequently may not have the contacts, skills or resources to influence relevant policies [34]. Moreover, they only represent thirteen low and lower-middle income countries. Secondly, once appropriate advocates are identified, they may need help in identifying the key target audiences for knowledge transfer and in identifying channels to overcome political barriers and influence those who are able to act [6]. In some countries, policy actors such as the WHO, UNICEF and other international agencies may play an important role in opening doors to national level policy makers and in promoting evidence-based policies and practice. Given this influence, the WHO and other international agencies should consider whether to raise the standards of evidence that they use in their advisory work to national governments and in their own choices of policy and programme recommendations. Not all international agencies, however, see themselves as having an advocacy role in changing clinical practice. For example, efforts made by the Magpie Trial investigators and others to persuade the President of the International Federation of Obstetricians and Gynaecologists that he and the organisation had potentially important roles in promoting the uptake of magnesium sulphate met with reluctance for himself or his organisation to interfere in the clinical freedom of individual clinicians (Chalmers, personal communication 2005) [21].

Our findings highlight the importance placed by respondents on interactions between policy makers, researchers and other stakeholders in facilitating the uptake of research findings into policies. Other studies examining the process of transferring research findings to key policy actors have also suggested that this process should be interactive in order to maximise its effectiveness [6]. These interactions can be initiated by individual researchers or policy makers but systems level mechanisms, such as observatories that bring together the producers and users of research, may also be useful [3,4,34,35] and should be considered for low and middle income countries, as part of strengthening national health research systems [1]. The sharing of experiences between such units needs to be encouraged so as to identify generalisable lessons for research translation. Financial support to ensure that magnesium sulphate is licensed and imported or produced locally may also be necessary. Moreover, financial support may be important to eliminate out of pocket payments that are acting as barriers in some settings to accessing essential medical care such as magnesium sulphate for eclampsia and severe pre-eclampsia.

At the implementation level, training health care professionals in developing countries to provide appropriate care presents challenges that are similar to those encountered in high-income countries [36-38]. These challenges are even more important to address in low-income countries because of the more severe consequences of not doing so and a greater need to use scarce resources efficiently. Evidence-based, international guidelines that can be locally adapted, such as those provided in the WHO Reproductive Health Library [39], could provide a valuable basis for both developing and implementing appropriate national clinical policies and practice guidelines. Other interventions to improve the use of medicines in developing countries have been outlined elsewhere [40], and may assist in addressing delays in responding to new evidence on the effectiveness of drugs.

More broadly, it would seem sensible to organise a wide programme of support rather than multiple one-off efforts for essential medicines in maternal and child health. Such a programme could provide an ongoing framework and support for ensuring that important research findings, such as those of the Magpie Trial, are translated into appropriate policies and practice.

## Competing interests

MA has previously carried out short-term pharmacoeconomic projects for the National Insurance Service and the Norwegian Medicines Agency. In 1997–99 he worked for a private company, Brevreklame, doing market research for pharmaceutical firms in Norway. LD was the clinical coordinator of the Collaborative Eclampsia and Magpie trials.

## Authors' contributions

MA, SI, MT, SL and ADO planned the group interview, observation and survey of the Magpie Collaborative Group with advice from LD. MA, SI, MT and SL undertook the interview, observations and survey. MA and SI coded and analysed the results of the survey. MA, EJP, ATD and ADO planned the survey of drug information officers with advice from SL, LD and MZ. EJP and ATD undertook the pilot survey. MA and SI prepared the first draft of the report. All of the authors commented on successive drafts.

## Pre-publication history

The pre-publication history for this paper can be accessed here:


